# A Signature Based on Costimulatory Molecules for the Assessment of Prognosis and Immune Characteristics in Patients With Stomach Adenocarcinoma

**DOI:** 10.3389/fimmu.2022.928742

**Published:** 2022-07-22

**Authors:** Bangjie Chen, Yong Yao, Deshen Mao, Conghan Li, Xingyu Wang, Shuyan Sheng, Lizhi Zhang, Xinyi Wang, Sanwei Chen, Wentao Xu, Jianyi Deng, Chenyu Sun, Qin Zhou, Scott Lowe, Rachel Bentley, Wei Shao, Haiwen Li

**Affiliations:** ^1^ First Clinical Medical College (First Affiliated Hospital), Anhui Medical University, Hefei, China; ^2^ School of Life Sciences, Anhui Medical University, Hefei, China; ^3^ School of Basic Medicine, Anhui Medical University, Hefei, China; ^4^ AMITA Health Saint Joseph Hospital Chicago, University of Illinois Chicago, Chicago, IL, United States; ^5^ Radiation Oncology, Mayo Clinic, Rochester, MN, United States; ^6^ Medical College, Kansas City University, Kansas, MO, United States; ^7^ Department of Gastroenterology, the First Affiliated Hospital of Zhengzhou University, Zhengzhou, China; ^8^ Third Affiliated Hospital (Hefei First People’s Hospital), Anhui Medical University, Hefei, China

**Keywords:** costimulatory molecule, STAD, prognostic signature, nomogram, immunotherapy

## Abstract

Although costimulatory molecules have been shown to boost antitumor immune responses, their significance in stomach adenocarcinoma (STAD) remains unknown. The purpose of this study was to examine the gene expression patterns of costimulatory molecule genes in patients with STAD and develop a predictive signature to aid in therapy selection and outcome prediction. We used 60 costimulatory family genes from prior research to conduct the first complete costimulatory molecular analysis in patients with STAD. In the two study groups, consensus clustering analysis based on these 60 genes indicated unique distribution patterns and prognostic differences. Using the least absolute shrinkage and selection operator and Cox regression analysis, we identified nine costimulatory molecular gene pairs (CMGPs) with prognostic value. With these nine CMGPs, we were able to develop a costimulatory molecule-related prognostic signature that performed well in an external dataset. For the patients with STAD, the signature was proven to be a risk factor independent of the clinical characteristics, indicating that this signature may be employed in conjunction with clinical considerations. A further connection between the signature and immunotherapy response was discovered. The patients with high mutation rates, an abundance of infiltrating immune cells, and an immunosuppressive milieu were classified as high-risk patients. It is possible that these high-risk patients have a better prognosis for immunotherapy since they have higher cytolytic activity scores and immunophenoscores of CTLA4 and PD-L1/PD-L2 blockers. Therefore, our signature may help clinicians in assessing patient prognosis and developing treatment plans.

## Introduction

Gastric cancer (GC) is a global public health burden, affecting more than one million individuals and causing an estimated 769,000 deaths (equating to 1 in every 13 deaths globally) each year (1). It is the fourth leading cause of cancer mortality globally (1) despite breakthroughs in surgical methods, radiation, chemotherapy, and neoadjuvant treatment. GC has high molecular and phenotypic diversity. Endoscopic resection is the most common treatment for early GC and surgery for advanced or intermediate staged GC (2). Owing to the low rate of early detection, surgery as a first-line treatment frequently does not yield the desired outcome (3). The combination of immunotherapy and chemotherapy is considered a powerful treatment for advanced GC (4). Patients with GC and/or gastroesophageal junction cancer may respond to targeted treatment based on four molecular indicators: T-DM1 and PD-L1 expression is necessary for trastuzumab and trastuzumab deruxtecan and MSI and HER2 positivity for pembrolizumab (5). Therefore, finding novel biomarkers that can predict patient survival and responsiveness to targeted medicines or immunotherapies is critical.

Multiple clinical studies are combining immune checkpoint inhibition (ICI) therapy with conventional chemotherapy. Success has been documented in non-small- and small-cell lung carcinomas (6, 7), as well as in esophageal (8), urothelial (9), gastric (10), and head and neck malignancies (11). However, the objective response rate is poor, and some patients develop drug resistance and disease progression after ICI therapy. In addition, immunotherapies, such as vaccine therapy and genome editing, are widely used in patients with GC. Initial attempts of other immunotherapies, such as CAR-T therapy, have prompted the advancement of immunotherapy in GC (12). However, the high heterogeneity of GC makes screening for typical biomarkers difficult. Identification of more biomarkers and mobilization of tumor-reactive lymphocytes from patients in a rapid and accurate manner should be the focus of future studies (13). Through genomic profile analysis, The Cancer Genome Atlas (TCGA) identified four distinct subtypes of stomach adenocarcinoma (STAD) in 2014: microsatellite unstable (MSI), genomically stable, Epstein–Barr virus-positive, and chromosomally unstable cancers (14). Consequently, it may be possible to develop new concepts for more precise molecular subtypes and tailored therapies if representative gene sets are selected for tumor classification and if prediction models are constructed. A number of studies have confirmed that costimulatory molecules are closely related to pathological tumor angiogenesis (15–17). Given the importance of angiogenesis in GC, using costimulatory molecules to enable efficient risk classification and identify possible targets for tailored therapeutic approaches appears to be extremely promising. Previous studies have shown that costimulatory molecules have therapeutic potential in various cancers (18). T-cell activation and proliferation are regulated by costimulatory molecules, making them potential targets for the development of novel ICI therapy. Immunological tumor milieu regulation may also be one of these functions (19, 20). However, it is unclear what specific roles these costimulatory chemicals play in the pathogenesis of GC.

In this study, we examined the expression patterns and prognostic significance of costimulatory molecular gene pairs (CMGPs) in patients with STAD. We then created and verified a predictive signature and nomogram for these patients. In STAD, a risk model based on CMGPs showed promise in predicting survival. Furthermore, a nomogram combining a risk model with clinical parameters effectively predicted the prognosis of patients with STAD. Finally, we assessed the potential response to immunotherapy and chemotherapy among several patient groups classified using the CMGP-based signature. Notably, similar data mining, processing, and model building have been achieved in renal cell carcinoma (21, 22), prostate cancer (23), hepatocellular carcinoma (24), etc.

## Materials and Methods

### Data Collection

The RNA-seq and important clinical features of patients with STAD were downloaded as the modeling cohort from the TCGA database (https://portal.gdc.cancer.gov/), and the dataset was randomly divided into a training cohort and an internal test cohort at a 7:3 ratio. Furthermore, we used data from the Gene Expression Omnibus (GEO) (GEO-GSE15459) database (https://www.ncbi.nlm.nih.gov/geo) as the external validation cohort. In tumor immunotherapy, the tumor immune checkpoint pathways PD-L1/PD-1 and CD86/CTLA4 belong to the B7-CD28 family, and other costimulatory pathways mainly originate from the tumor necrosis factor (TNF) family. At present, 13 molecules are classified as members of the B7-CD28 family, including 8 molecules belonging to the B7 family (CD80, CD86, PD-L1, PD-L2, ICOSLG, B7-H3, B7x, and HHLA2) and 5 molecules belonging to the CD28 family (CD28, CTLA4, ICOS, PD-1, and TMIGD2). The TNF family consists of the TNF ligand superfamily (TNFSF) and the TNF receptor superfamily (TNFRSF) with 47 molecules. Among them, 18 ligands are members of the TNFSF, and the other 29 receptors are members of the TNFRSF. Herein, we identified 60 costimulatory molecule genes (CMGs) from the study by Zhang et al. (25) and downloaded them for further analyses. All the data used in our study are publicly available.

### Consensus Clustering Analysis

Consensus clustering was used to further investigate the roles and prognostic importance of the costimulatory molecules in STAD using the “ConsensusClusterPlus” R program (26). The clustering score for the cumulative distribution function curve determined the optimal cluster number. The algorithm first subsampled a proportion of items and features from the data matrix. Thereafter, each subsample was divided into, at most, k groups using an agglomerative hierarchical clustering algorithm. This process was repeated for a specified number of times. The pairwise consensus value, defined as “the running proportion of clusters where two items are together (group),” was computed for each k and stored in the consistency matrix. Agglomerative hierarchical clustering was performed using a consensus value of 1 for each k, which was then pruned into k groups, called consensus clusters.

### Comparison of Immune Cell Infiltration and Tumor Microenvironment Between the STAD Subtypes

We calculated the abundance of eight immune cells and two stromal cells using the “MCPcounter” R package (27). The score indicated the degree of infiltration to the immune microenvironment. The tumor microenvironment (TME) scores (stromal, immune, and estimate scores) for the total STAD cohort were calculated using the “ESTIMATE” package (28).

### Functional Analyses

The “Limma” (29) R software was used to identify genes whose expression was different between the two groups. The “GSVA” (30) R package was used to reveal how the signaling pathways differed between the two clusters *via* a gene set variation analysis.

### CMGP-Based Prognostic Model Construction and Validation Using the Least Absolute Shrinkage and Selection Operator and Cox Regression Analysis

Sixty costimulatory molecules were pairwise aligned, and 3,540 permutations could be formed according to random permutations; the expression quantity of the gene pairs in each sample in the TCGA database was examined. When the former gene was more highly expressed than the latter, it was labeled as 1; the reverse was marked as 0. When a gene pair was >20% scaled to 1 or 0, it was eliminated. The CMGPs were obtained using pairwise comparisons and gene expression analyses in the same patient, which avoided batch effects associated with multiple platforms and eliminated the need to scale and normalize the data. The CMGPs linked with prognosis were identified using univariate Cox regression analysis (*p* < 0.001). The least absolute shrinkage and selection operator (LASSO)–Cox regression model was built using the prognostic-associated CMGPs derived from the univariate Cox regression analysis. We then used the LASSO method with penalty parameter tweaking, conducted *via* 10-fold cross-validation, to exclude the CMGPs that may be substantially associated with other CMGPs. A subset of CMGPs was identified by decreasing the regression coefficient with a penalty proportional to their size. For future multivariate Cox regression analysis, the CMGPs with nonzero regression coefficients were maintained. We compared the predictive CMGP values with the regression coefficients from the multivariate Cox proportional hazard regression analysis (β) to create a risk score model. For the LASSO regression analysis of the prognostic CMGPs, the “glmnet” (31) R package was employed. The median risk score was used to divide the patients into high- and low-risk categories. The “survminer” (32) and “timeROC” (33) R packages were used to create the Kaplan–Meier survival and receiver operating characteristic (ROC) curves of the risk score, which were used to estimate the model’s predictive power. Clinical usefulness was assessed using decision curve analysis (DCA). To compare the two groups in terms of the survival curve, we used the log-rank test and set the statistical significance level at *p* < 0.05. The GSE15459 cohort was used for external validation, whereas the TCGA cohort was split at a 7:3 ratio into a training cohort and an internal validation cohort.

### Correlation Analysis Between the Prognostic Model and TME

The TME scores (stromal score, immune score, estimate score, and tumor purity) were calculated using the “ESTIMATE” package (28), and gene expression data were utilized to determine the infiltrating stromal or immune cells in the tumor tissues. Additionally, we used the Wilcoxon test to compare the four types of scores between the two groups, as well as the Pearson correlation test to examine the link between the risk score and the four TME scores. According to the TCGA database, tumor mutation burden (TMB) was defined as the total number of somatic gene coding mistakes, base substitutions, insertions, and deletions detected per million bases (34). We examined the association between the TMB and odds of survival.

### Gene Set Enrichment Analysis Based on the GO and KEGG Datasets

We used cp.kegg.v7.1.symbols.gmt and go.v7.4.symbols.gmt in the “cluster profiler” package (35) to analyze the highly expressed genes both in the low- and high-risk groups as a reference gene set and the function gesaplots to plot the results, filtering significantly enriched pathways with *p* < 0.05 as a threshold (FDR < 0.25).

### Nomogram Construction and Evaluation

We used the “RMS” (36) R package to integrate variables, such as age, tumor stage, and risk score, and the Cox method to establish a nomogram to evaluate STAD prognosis. The “timeROC” (33) R program was used to assess the prognostic performance of the nomogram model based on the time-dependent ROC curves. The concordance index (C-index) was used to measure the likelihood of the projected result matching the actual result. The 45 dotted line indicated the best prediction. Calibration curves were generated to test the discriminative ability of the nomogram.

### Chemical Reaction Prediction

We predicted the treatment response for each sample using the world’s largest publicly available pharmacogenomics database, Genomics of Drug Sensitivity in Cancer (https://www.cancerrxgene.org/), and the “pRRophetic” package (37).

### Statistical Analyses

To compare two variables, we used the *t*-test or Wilcoxon test. To assess survival differences, we performed the Kaplan–Meier method and log-rank tests(two-stage test was used when curves crossed (38)). The predictive impact of the CMGs was assessed using univariate and multivariate Cox regression models. To assess variations in the distribution of the clinical variables among the patients with STAD, we performed Pearson’s chi-squared test. The R software was used to perform all statistical analyses in this investigation. The statistical significance level was set at *p* < 0.05.

## Results

### Cluster Analysis Based on the CMG Expression Profiles

**Figure 1:**
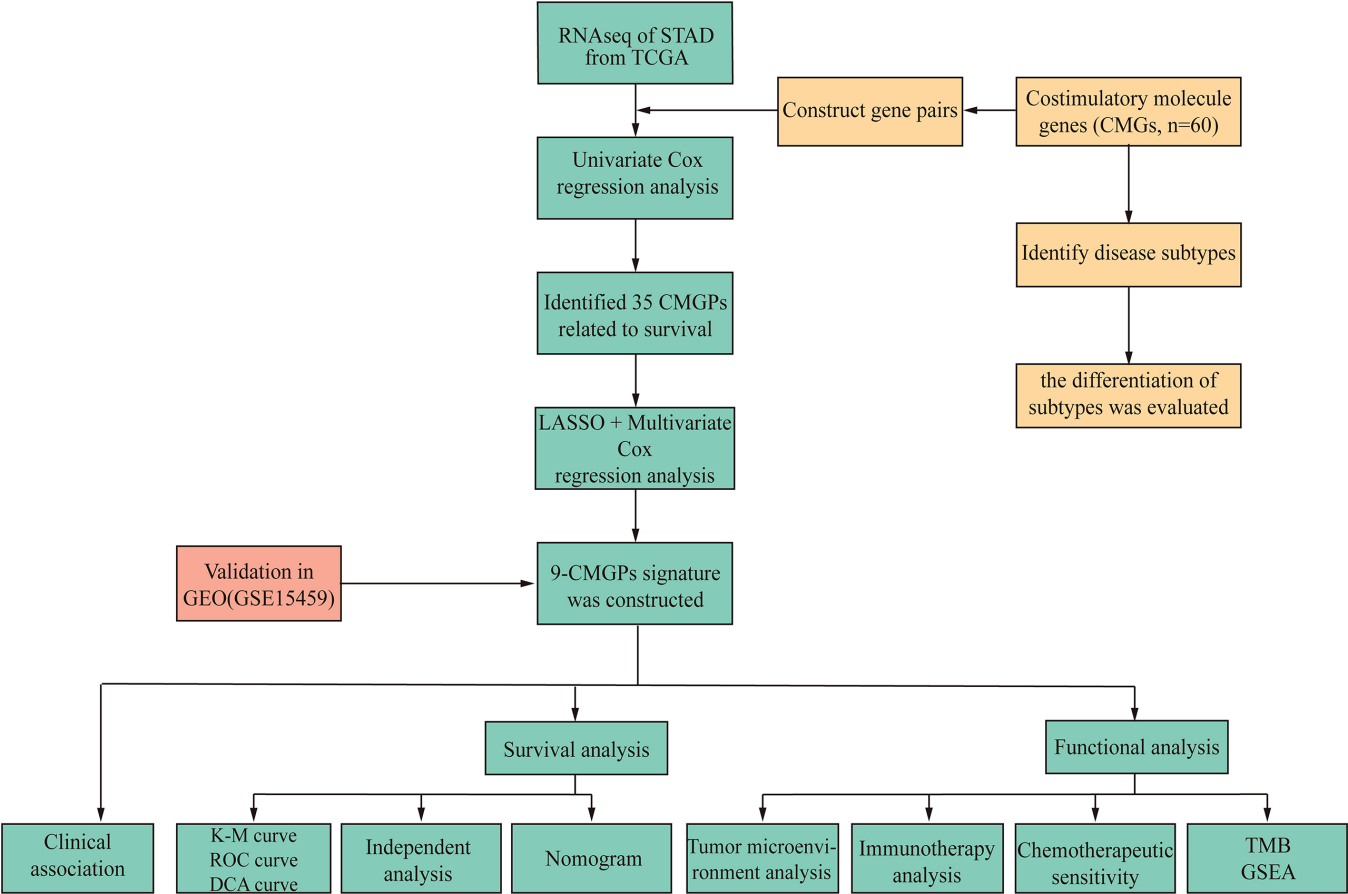
Flowchart of the data analysis.

**Figure 2:**
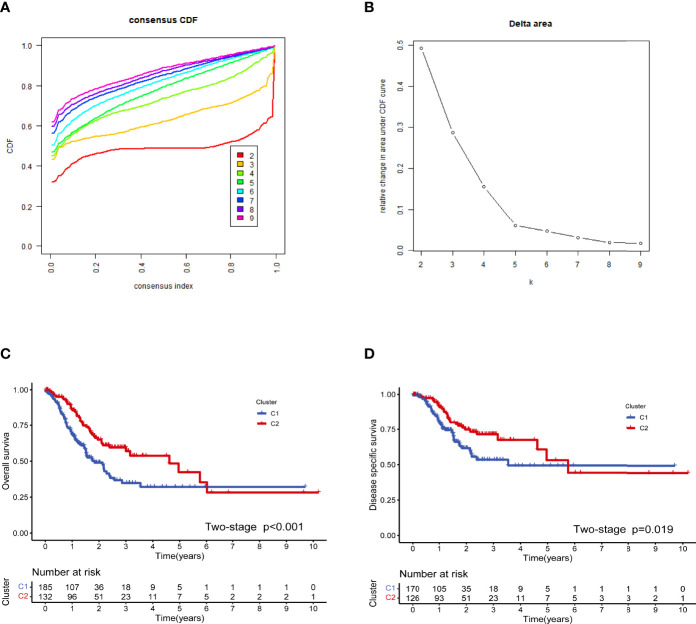
Cluster analysis based on the costimulatory molecule gene (CMG) expression profiles. The optimal value for consensus clustering **(A, B)** was found to be k = 2. Kaplan–Meier curve for the disease-free survival in the stomach adenocarcinoma (STAD) group **(C)**. Kaplan–Meier curve for the total survival in the STAD group **(D)**.

The workflow of this study is illustrated in **Figure 1**. We used a consensus clustering technique to stratify the patients with STAD to determine the overall prognostic value of the genes. We discovered that a k value of 2 appeared to be a more stable number between values of 2 and 9 (**Figures 2A, B**). The Kaplan–Meier curves revealed that the patients in cluster 1 showed worse overall survival (OS) (**Figure 2C**) and disease-specific survival (DSS) (**Figure 2D**) than did the patients in cluster 2 in the two molecular subtypes. There is a partial crossover at the end of the survival curve, suggesting that other factors may have an impact on the survival outcome. However, the crossover is located at the end of the curve, and the number of patients is small; thus, it is difficult to analyze hierarchically. Referring to relevant statistical literature, when the survival curves are crossed, the log-rank test is no longer used, but the two-stage test should be used (38). The *p*-value of the two-stage test is still less than 0.05, indicating that costimulatory molecular genes are indeed an important factor affecting survival, and the overall survival of C2 patients is longer than C1.

### Immune Status in the Two Clusters

**Figure 3:**
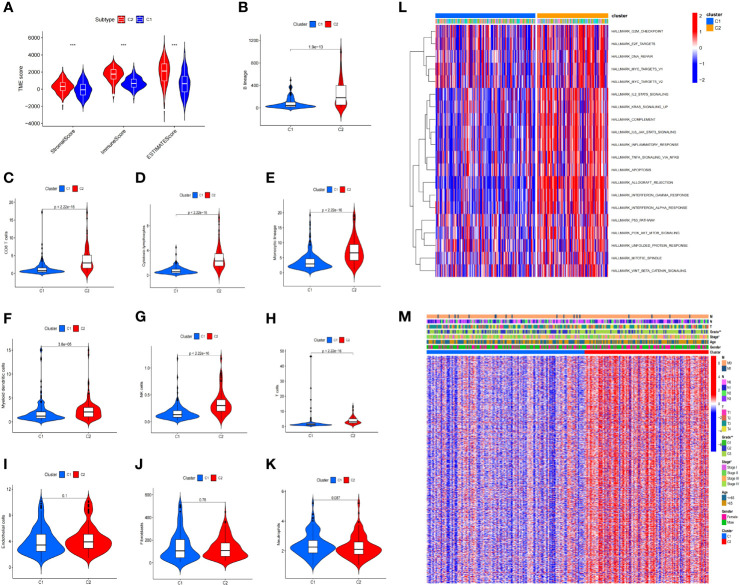
Immune difference between the two clusters. The patients in cluster 2 had substantially higher immunological, estimation, and stromal scores than those in cluster 1 **(A)**. Furthermore, the abundance of B lineage **(B)**, CD8^+^ T cells **(C)**, cytotoxic lymphocytes **(D)**, monocytic lineage **(E)**, myeloid dendritic cells **(F)**, NK cells **(G)**, and T cells **(H)** was significantly higher in cluster 2 than in cluster 1, while no significant difference was found for the abundance of endothelial cells **(I)**, fibroblasts **(J)**, and neutrophils **(K)**. Gene set variation analysis of the two clusters **(L)**. Differences in the clinicopathological characteristics between the two clusters **(M)** (^*^ *p* < 0.05, ^**^ *p* < 0.01, ***p < 0.001).

Immunological differences between the two molecular subtypes have been investigated in previous immune studies. According to the estimation algorithm, the patients in cluster 2 had substantially higher immune scores (*p* < 0.001), estimated scores (*p* < 0.001), and stromal scores (*p* < 0.001) than those in cluster 1 (**Figure 3A**). In addition, the abundance of B lineage (*p* = 1.9e−13) (**Figure 3B**), CD8^+^ T cells (*p* < 2.22e−16) (**Figure 3C**), cytotoxic lymphocytes (*p* < 2.22e−16) (**Figure 3D**), monocyte lineage (*p* < 2.22e−16) (**Figure 3E**), myeloid dendritic cells (*p* = 3.8e−05) (**Figure 3F**), NK cells (*p* < 2.22e−16) (**Figure 3G**), and T cells (*p* < 2.22e−16) (**Figure 3H**) was significantly higher in the patients in cluster 2 than in those in cluster 1; meanwhile, no significant difference was detected with respect to the abundance of endothelial cells (*p* = 0.1) (**Figure 3I**), fibroblasts (*p* = 0.78) (**Figure 3J**), and neutrophils (*p* = 0.087) (**Figure 3K**).

### Differentially Expressed Genes and Functional Analyses

Differentially expressed genes (DEGs) were identified between the two clusters, and functional investigations were performed to investigate the underlying signaling processes. Cluster 2 had 893 DEGs, 126 of which were downregulated and 767 were upregulated, compared with cluster 1. To evaluate the relationship between the enriched pathways and the prognosis of the patients with STAD, we utilized GSVA analysis to analyze the relative expression differences in the pathways in the two clusters. The heatmap in **Figure 3L** shows a number of differentially expressed pathways enhanced by the GSVA analysis.

### Distribution of the Clinical Features in the Two Clusters

We examined the distribution of the clinical characteristics in the two clusters. The analysis showed that there was a significant difference in grade and stage, but none in age, sex, or other clinical features (**Figure 3M**).

### Development of a Risk Model Based on the CMGs in the TCGA Training Cohort

**Figure 4:**
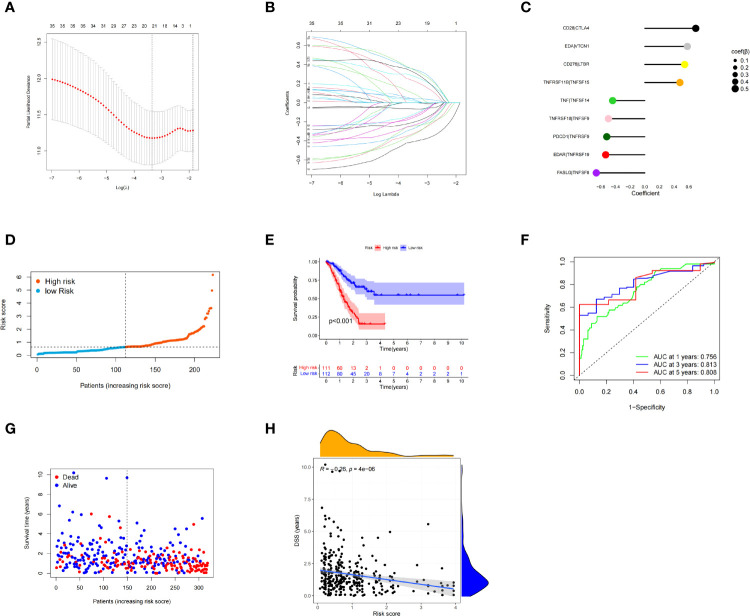
Multivariate least absolute shrinkage and selection operator (LASSO) regression analysis. Nine costimulatory molecular gene pairs (CMGPs) were identified using Cox regression analysis to create the prognostic signature **(A, B)**. Coefficient of the nine CMGPs **(C)**. The risk score of each patient was generated, and the 223 patients were divided into high- and low-risk groups according to the median risk score (0.6378) **(D)**. Survival curves in the high- and low-risk groups **(E)**. Time-dependent receiver operating characteristic (ROC) curve of the risk model **(F)**. The number of deaths increased as the risk score increased; this trend was more obvious with the increase in the risk score, especially in the very high-risk population. There were significant differences in the clinical outcomes at the symmetrical positions of the left and right sides of the median value, especially when the risk score was <100 and >200 **(G)**. Disease-specific survival (DSS) and risk score correlation **(H)**.

The univariate Cox proportional hazard regression analysis identified 35 CMGPs that were related to survival. After LASSO regression (**Figures 4A, B**) and multivariate Cox regression (**Figure 4C**) analyses, nine CMGPs were selected and utilized to build a prognostic signature as follows: Risk score = (0.54369 *`CD276|LTBR`) + (0.69502 *`CD28|CTLA4`) + (0.58032 *`EDA|VTCN1`) + (−0.53341 *`EDAR|TNFRSF19`) + (−0.66137 *`FASLG|TNFSF8`) + (−0.51890 *`PDCD1|TNFRSF9`) + (−0.43837 *`TNF|TNFSF14`) + (0.47905 *`TNFRSF11B|TNFSF15`) + (−0.49658 *`TNFRSF18|TNFSF9`). We used this method to determine each patient’s risk score and divided 223 individuals into high- and low-risk groups based on the median value of the risk score (0.6378) (**Figure 4D**). The OS of the high-risk group was lower than that of the low-risk group (*p* < 0.001), as evidenced by the survival curve (**Figure 4E**). The risk scores that predicted OS at 1, 3, and 5 years had area under the curve of ROC (AUC) values of 0.756, 0.813, and 0.808, respectively (**Figure 4F**). The frequency of fatalities increased as the risk score increased, and this trend was more obvious with the increase in the risk score, especially in the very high-risk population (**Figure 4G**); meanwhile, the DSS rate decreased (**Figure 4H**). These preliminary findings suggest that stratifying prognosis based on the risk score is useful.

### Internal Validation of the Prognostic Model in the TCGA Test Cohort

**Figure 5:**
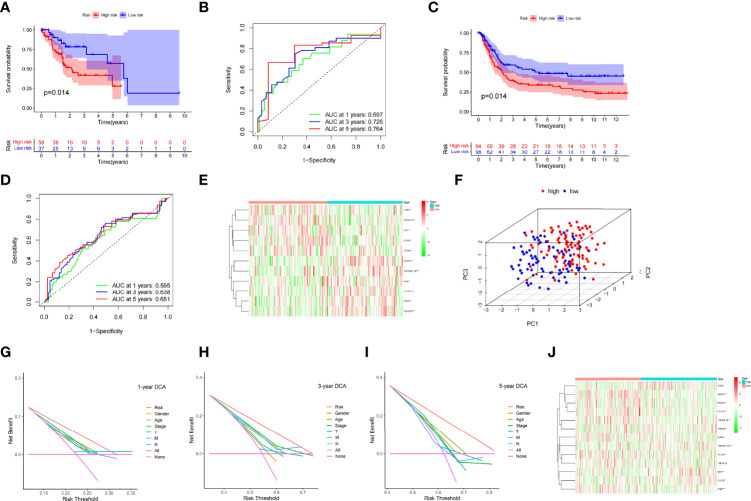
Kaplan–Meier curves in the TCGA test cohort **(A)** and the GEO validation cohort **(C)**. TCGA test cohort **(B)** and GEO validation cohort **(C)** time-dependent receiver operating characteristic curves for the sensitivity and specificity of the prognosis assessment **(D)**. Gene set variation analysis of the GEO validation cohort (the unit of color bars is log2 (actual expression) +1) **(E)**. In the TCGA dataset, principal component analysis was used to evaluate the distribution patterns of the high- and low-risk patients **(F)**. Prognostic signature decision curve analysis at 1 **(G)**, 3 **(H)**, and 5 years **(I)**. Gene set variation analysis of the entire TCGA cohort (the unit of color bars is log2 (actual expression) +1) **(J)** (^*^ *p* < 0.05, ^**^ *p* < 0.01, ^***^ *p* < 0.001).

The prognosis of the high-risk group was considerably poorer than that of the low-risk group in the TCGA test cohort (*n* = 95) (**Figure 5A**). The risk scores that predicted OS at 1, 3, and 5 years had AUC values of 0.697, 0.726, and 0.764, respectively (**Figure 5B**). The risk score had good accuracy in predicting STAD prognosis, based on the findings of the internal validation.

### External Validation of the Prognostic Model in the GEO Cohort

The GSE15459 cohort from the GEO database was used as an external dataset to validate the prognostic model because of its large sample size (*n* = 192) and complete clinical data. The risk score for each patient in the cohort was determined using a prior method, and the patients were classified into high- or low-risk groups based on the unified cutoff value (0.6378). The high-risk group had significantly lower OS rates than the low-risk group, consistent with earlier research findings (**Figure 5C**). AUC values of 0.595, 0.638, and 0.651 were found in the risk score that predicted OS at 1, 3, and 5 years, respectively (**Figure 5D**). The heatmap in **Figure 5E** shows the expression patterns of 11 CMGs in the patients with varied risk levels. The external validation revealed that the prognostic model created had a wide range of applications and was very stable in predicting STAD prognosis.

### Prognostic Assessment of the Prognostic Model in the Entire TCGA Cohort

For analysis, we included all the study items in a single TCGA cohort (*n* = 318). In the high- and low-risk groups, principal component analysis revealed different distribution patterns (**Figure 5F**). In the TCGA cohort, DCA of the nomogram revealed that the nomogram model had a good net benefit for 1-year (**Figure 5G**), 3-year (**Figure 5H**), and 5-year (**Figure 5I**) OS. Accordingly, the nomogram based on the risk score may be utilized as an effective tool for predicting patient prognosis in clinical practice. The heatmap in **Figure 5J** depicts the expression patterns of 13 CMGs in the patients with varying risk ratings. The risk score may also be used to predict prognosis independently, as revealed in the univariate (**Supplementary Figure 1H**) and multivariate regression analyses (**Supplementary Figure 1I**). These findings demonstrated the dependability and consistency of the predictive signature.

### Clinical Correlation Analysis of the Prognostic Model

In the TCGA cohort, we examined sex, age, grade, pathological stage, T stage, M stage, and N stage to determine whether there was a link between immunotyping and common clinical characteristics. The results suggested that our prognostic signature was not significantly associated with the clinical factors in STAD (**Supplementary Figures 1A–G**).

### Relationship Between the Prognostic Model and Immune Infiltration

**Figure 6:**
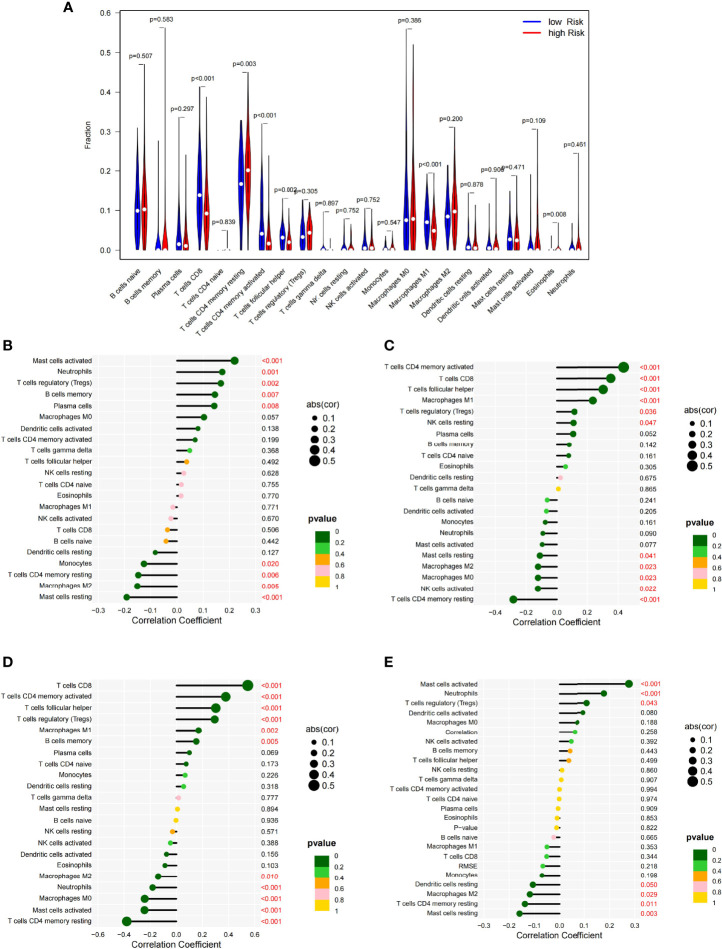
Vioplot of the absolute abundance of 22 immune infiltration cells between the high- and low-risk groups **(A)**. Correlation coefficients between the immune cells and CD28 **(B)**, CTLA4 **(C)**, PDCD1 **(D)**, and TNF **(E)**.

The degree of infiltration of the immune cells varied between the high- and low-risk groups. For example, the high-risk group had significantly more CD4 memory resting T cells (*p* = 0.003) and eosinophil infiltrates (*p* = 0.008) than the low-risk group; meanwhile, the low-risk group had significantly more CD8^+^ T cells (*p* < 0.001), CD4 memory-activated T cells (*p* < 0.001), follicular helper T cells (*p* = 0.002), and M1 macrophages (*p* < 0.001) than the high-risk group (**Figure 6A**). These findings suggested that the tumor immune microenvironment (TIME) was related to the risk score. The four genes [CD28 (**Figure 6B**), CTLA4 (**Figure 6C**), PDCD1 (**Figure 6D**), and TNF (**Figure 6E**)] in the model showed a strong correlation with some immune cells. These findings provide useful information for future research.

### Relationship Between the Prognostic Model and TME

**Figure 7:**
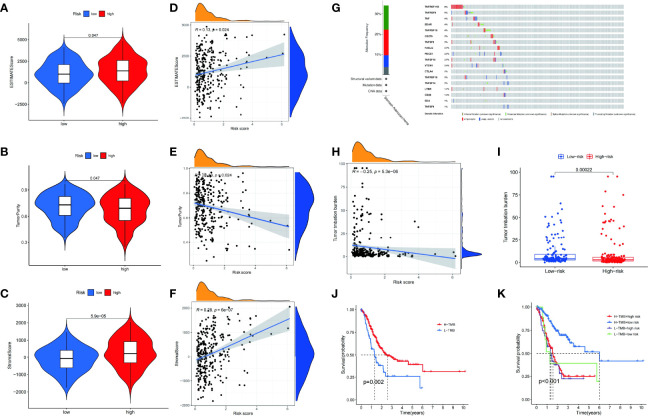
The estimate scores **(A)**, tumor purity **(B)**, and stromal scores **(C)** differed between the high- and low-risk patients. Spearman correlation analysis of the risk scores, estimate scores **(D)**, tumor purity **(E)**, and stromal scores **(F)**. Genetic mutations: types and frequencies **(G)**. Spearman correlation analysis of tumor mutation burden (TMB) and risk score **(H)**. Difference in TMB between the high- and low-risk patients **(I)**. Kaplan–Meier curve for the overall survival of the high- and low-TMB patients **(J)**. Kaplan–Meier curve for the overall survival of the high-TMB/high-risk, high-TMB/low-risk, low-TMB/high-risk, and low-TMB/low-risk patients **(K)**.

The patients with a lower estimate score (*p* = 0.047) (**Figure 7A**) and stromal score (*p* = 5.9e05) (**Figure 7B**) and greater tumor purity (*p* = 0.047) had a better prognosis (**Figure 7C**) than their counterparts. Patients with a lower immune score (*p* = 0.79) had no statistical significance. Pearson correlation analysis showed that the estimated score (*R* = 0.13, *p* = 0.024) (**Figure 7D**) and stromal score (*R* = 0.28, *p* = 6e−07) (**Figure 7F**) were positively correlated with the risk score, while tumor purity (*R* = -0.13, *p* = 0.024) was negatively correlated with the risk score (**Figure 7E**). In addition, no correlation was found between the immune score (*R* = −0.038, *p* = 0.49) and the risk score. Accordingly, the risk score can be used to analyze the TME.

### Differences in the Genomic Alterations Between the High- and Low-Risk Groups

Genomic mutations are closely associated with tumorigenesis. Therefore, the frequency of alterations in patients with STAD was studied. Among the CMGs, TNFRSF11B had the highest genetic alteration rate (**Figure 7G**). Furthermore, there was a significant difference in the TMB (*p* = 0.00022) between high- and low-risk groups (**Figure 7I**). Using Pearson correlation analysis, we also validated the clearly negative relationship between the risk score and TMB (**Figure 7H**). The high TMB group had a somewhat higher OS rate than the low TMB group (**Figure 7J**). The patients were divided into four groups based on their risk score and TMB. We found that the group with the highest TMB and lowest risk score had the best survival rate (**Figure 7K**).

### Gene Set Enrichment Analysis Between the Different Risk Groups

We performed gene set enrichment analysis of the various risk groups to identify probable molecular mechanisms for the prognostic model. The analysis revealed that the gene sets in the high-risk group were mostly abundant in receptor- or metastasis-related pathways, such as the KEGG (ECM–receptor interaction, complement and focal adhesion, coagulation cascades, neuroactive ligand–receptor interaction, and PPAR signaling pathway) (**Supplementary Figure 2A**) and GO pathways (behavior, cell matrix adhesion, cell substrate adhesion, circulatory system process, and external encapsulating structure organization) (**Supplementary Figure 2B**). Most of the pathways that presented a significant enrichment in the low-risk group were related to immunology, including the KEGG (autoimmune thyroid disease, graft versus host disease, antigen processing and presentation, allograft rejection, and proteasome) (**Supplementary Figure 2C**) and GO pathways (activation of immune response, adaptive immunological response based on immunoglobulin superfamily domain-based somatic recombination of immune receptors, B cell-mediated immunity and complement activation, and antigen receptor-mediated signaling pathway) (**Supplementary Figure 2D**). These findings provide important information for future research on the molecular mechanisms underlying STAD.

### Nomogram Construction and Validation

**Figure 8:**
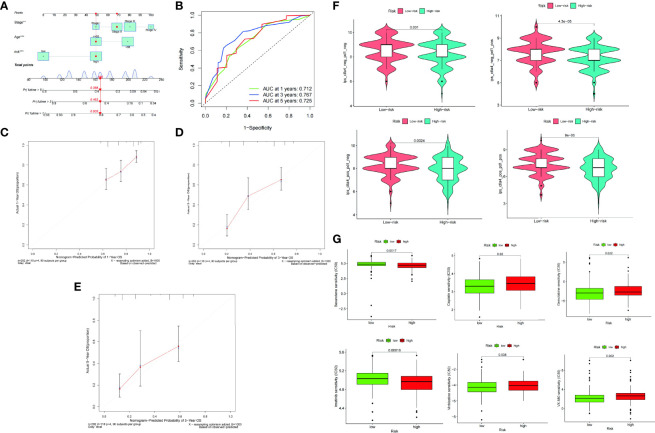
A nomogram was constructed on the basis of age, pathological stage, and risk score **(A)**. ROC curve of the nomogram at 1, 3, and 5 years **(B)**. Calibration plots of overall survival (OS) at 1 **(C)**, 3 **(D)**, and 5 years **(E)**. Differences in PD-1 and CTLA-4 therapy sensitivity between the low- and high-risk populations **(F)**. Differences in chemotherapy sensitivity between the low- and high-risk populations **(G)**.

A visual nomogram was created to produce a therapeutically useful tool to determine the prognosis of patients with STAD. The nomogram was built using a training set that predicted the OS. It incorporated age, pathological stage, and risk score as integrated clinicopathological variables (**Figure 8A**). The predictive value of the nomogram was assessed using ROC analysis and the C-index. In the TCGA dataset, the AUC values of the predictive value of the nomogram for 1-, 3-, and 5-year OS were 0.712, 0.767, and 0.725, respectively (**Figure 8B**). In terms of the 1-year (**Figure 8C**), 3-year (**Figure 8D**), and 5-year OS (**Figure 8E**) in the TCGA cohort, the calibration plots revealed a sustained concordance between the nomogram-projected probability and actual observation.

### Relationship Between the Risk Scores for Immunotherapy and Chemotherapy

The immunophenotype score was used to assess the ICI therapy response. In the comparison between the low- and high-risk groups, we discovered that the proportion of CTLA4 and PD1 was somewhat greater in the low-risk group than in the high-risk group (**Figure 8F**). The low-risk group was more sensitive to the chemotherapy drugs, such as cisplatin, gemcitabine, imatinib, vinblastine, and VX.680, than the high-risk group. In contrast, the high-risk group was more sensitive to bexarotene than the low-risk group (**Figure 8G**).

## Discussion

Currently, GC ranks fifth in incidence and fourth in mortality among cancer cases worldwide (1). Owing to the lack of early diagnosis, patients who are detected to have GC are mostly terminal patients who can only benefit slightly from surgical treatment (39). Complete tumor excision and lymph node dissection in combination with preoperative chemotherapy and postoperative adjuvant radiation and chemotherapy have been found to considerably enhance the postoperative survival time of patients with GC when the effects of surgery are restricted (40). With a few exceptions for patients with tumors of certain molecular subtypes, chemotherapy remains the mainstay of care (41). For patients with HER2+ tumors, trastuzumab, a HER2 targeting monoclonal antibody, is used for chemotherapy (42). PDL1 immunotherapy has recently emerged as a new treatment option for advanced GC because of advances in the research on the immune microenvironment of gastric tissues. Patients with a high MSI-H phenotype or high TMB (>10 mutations per megabase) may benefit from second-line treatment with pembrolizumab, a monoclonal anti-PD-1 antibody. Furthermore, patients with malignancies that express PD-L1 (combined positive score of 1) may also benefit from third-line treatment with this drug (43). Another anti-PD-1 antibody, nivolumab, improves OS as an advanced treatment for unselected patients with STAD and is combined with chemotherapy as a first-line treatment (44).

Clinical investigations have shown that ICI therapy is effective for STAD. Since the clinical use of this method, the identification of biomarkers for cancer diagnosis, efficacy, and prognosis has become a top priority in oncology immunotherapy research. The regulation of tumor immunity relies heavily on costimulatory molecules (45, 46). Monoclonal antibodies that target the PD-1/PD-L1 (B7-H1) or B7-2/CTLA-4 pathways have been shown to be promising in promoting long-term tumor regression in a range of cancers (47, 48). Costimulatory chemicals are responsible for all the therapeutic targets. However, there are few studies on the role of CMGs in the prognosis of STAD.

We obtained 60 members of the B7-CD28 and TNF families from patients with STAD in our study. To investigate the expression level and prognostic significance of the costimulatory molecules in STAD, we selected nine CMGPs (CD276|LTBR, CD28|CTLA4, EDA|VTCN1, EDAR|TNFRSF19, FASLG|TNFSF8, PDCD1|TNFRSF9, TNF|TNFSF14, TNFRSF11B|TNFSF15, and TNFRSF18|TNFSF9). The B7-CD28 family includes B7-H3 (CD276), an essential immunological checkpoint. B7-H3 is a protein produced by antigen-presenting cells and is involved in the suppression of T-cell activity. More importantly, it is overexpressed in a variety of human solid tumors and is often associated with poor prognosis among patients (49). The importance of the members of the B7-CD28 family and their ligands in immune activity has been demonstrated. However, many parts of CD28 biological activity remain unknown, and its translation into immunomodulatory treatments is uneven (50). TNF superfamily ligands have a wide range of biological activities, including cell death, survival, and proliferation, making them ideal therapeutic targets for cancer immunotherapy (51). Several members of the TNF family investigated in our study play pivotal roles in the immunotherapy of multiple cancers. However, these costimulatory molecules are novel and require further investigation in patients with STAD.

With the advancement of immunotherapy, it is critical to find biomarkers and select the most sensitive individuals to increase immunotherapy response rates. To investigate the overall prognostic value, we used a consensus clustering approach based on the 60 CMGs. According to the Kaplan–Meier curves, the patients in cluster 2 had a worse prognosis. These patients also had a high concentration of immune-related pathways, indicating that CMGPs are closely associated with the TIME. Patients classified under cluster 2 may have a poorer prognosis owing to immune system weakness or a limited immunological response.

Risk profiles based on CMGs might provide fresh insights into the clinical care of patients with STAD. In colorectal cancer (52) and lung adenocarcinoma, risk signatures based on CMGs have been developed (25). All these prognostic markers have been shown to be accurate and perform well. However, we were the first to develop a risk profile for patients with STAD based on CMGs. The performance of our prognostic signature was tested using the TCGA and E-MTAB-3267 datasets, both of which yielded positive results. We also discovered that the predictive signature was strongly linked to the clinical parameters, suggesting that it may be used as a complement to help guide treatment. We also selected genes that were substantially associated with the risk score of our prognostic signature, and the functional enrichment analysis revealed that T-cell homeostasis and NF-B signaling were enriched.

To further determine the links between our signature and the TIME, we analyzed immune cell infiltration and tumor mutation patterns in the high- and low-risk groups. In our analysis, the high-risk patients exhibited a much greater immune cell infiltration than did the low-risk patients. In addition, we found that the number of immunosuppressive cells, such as gamma delta T cells, MDSCs, monocytes, immature dendritic cells, macrophages, plasmacytoid dendritic cells, T follicular helper cells, and regulatory T cells, was larger in the high-risk patients than in the low-risk patients, indicating the presence of an immunosuppressive microenvironment. Tumor cells use an immunosuppressive microenvironment to evade immune responses and accelerate disease development. Understanding the immunological microenvironment of each patient will help identify patients who are more likely to respond to immunotherapy and enhance treatment response rates when combined with innovative treatment options.

TMB generally refers to the number of somatic non-synonymous mutations per megabase pair in a specific genomic region. It can indirectly reflect the ability and degree of tumor production of neoantigens and has been proven to predict the efficacy of immunotherapy for a variety of tumors (34, 53). Tumor-specific mutated genes can produce new proteins that are delivered by the major histocompatibility complex as well as their degradation products. They are present on the surface of tumor cells to form tumor neoantigens, which are then recognized by activated CD8^+^ T cells, thereby triggering tumor-targeted immune responses. Therefore, tumor gene mutations are considered the premise of antitumor immunotherapy (54). In recent years, an increasing number of studies have confirmed that tumors with higher TMB have higher neoantigen loads and are more likely to benefit from ICI therapy (55). To a certain extent, TMB reflects DNA repair damage in tumor cells and is closely related to the ability to generate tumor neoantigens (56). In 2014, TMB was first confirmed to correlate with the efficacy of the CTLA-4 antibody in the treatment of malignant melanoma (57). In 2015, tissue TMB (tTMB) was shown to be associated with the efficacy of PD-1 antibody treatment in patients with non-small-cell lung cancer (58, 59). A meta-analysis conducted in 2017 found that tTMB had a significant predictive effect on the efficacy of immunotherapy for 27 tumor types. There was a significant correlation between tTMB and ORR (*p* < 0.001), suggesting that tTMB is strongly correlated with the efficacy of PD-1/PDL1 antibodies (60). In this study, we found that the high-risk patients had a higher TMB than the low-risk patients, which may boost immunogenicity and result in a higher immunotherapy response rate. However, clinical trials in actual clinical settings are required to corroborate the above-mentioned outcomes.

This study had certain limitations. The data for this study were gathered retrospectively from public sources. The clinical indicators evaluated in this research were incomplete because of the limited number of datasets, including prognostic data for patients with STAD. Calculating the value of a prognostic signature requires actual prognostic information from patients with STAD. The genes were also limited to costimulatory molecules in this study, although the immunological TME was highly spatially heterogeneous. Consequently, the potency of the predictive signature is restricted. Furthermore, no evidence of CMG expression in the patients with STAD following immunotherapy was observed. Consequently, the risk signature utilized to evaluate immunotherapy response was indirect. Further prospective trials of immunotherapy in patients with STAD are needed to determine the therapeutic applicability of our signature.

In conclusion, we performed the first comprehensive study of costimulatory molecules in patients with STAD and identified nine pairs of genes with prognostic and diagnostic values. We created a costimulatory molecular-based prediction signature for patients with STAD and investigated its molecular underpinnings. With the use of our prognostic signature, the patients with a high mutation frequency, a large quantity of immune cell infiltration, and an immunosuppressive milieu were classified as high-risk patients. Taken together, our signature may help doctors in predicting the prognosis and selecting appropriate therapy for patients with STAD.

## Data Availability Statement

The datasets generated during and/or analysed during the current study are available in The Cancer Genome Atlas (TCGA) database (https://portal.gdc.cancer.gov/), Gene Expression Omnibus (GEO-GSE15459) database (http://www.ncbi.nlm. nih.gov/geo) and Genomics of Drug Sensitivity in Cancer (GDSC) (https://www.cancerrxgene.org/). The original contributions presented in the study are included in the article/**Supplementary Material**. Further inquiries can be directed to the corresponding authors.

## Author Contributions

All authors contributed to the study conception and design. HL, WS, and BC contributed to the conception of the study. BC, DM, CL, QZ, JD, SL, and RB performed the data analyses. BC, YY, XingW, SS, LZ, XinW, SC, WX, CS, QZ, SL, and RB contributed significantly in writing the manuscript. All authors contributed to the article and approved the submitted version.

## Funding

This study was supported by the Scientific Research Cooperation Project Between Anhui Medical University and Hefei First People’s Hospital (K202003, chaired by HL), the National-Level Innovation Training Program for Chinese College Students of the Ministry of Education of the People’s Republic of China (202110366010, chaired by BC) and the Research Level Improvement Project of Anhui Medical University (2021xkjT001, chaired by WS).

## Conflict of Interest

The authors declare that the research was conducted in the absence of any commercial or financial relationships that could be construed as a potential conflict of interest.

## Publisher’s Note

All claims expressed in this article are solely those of the authors and do not necessarily represent those of their affiliated organizations, or those of the publisher, the editors and the reviewers. Any product that may be evaluated in this article, or claim that may be made by its manufacturer, is not guaranteed or endorsed by the publisher.

## Supplementary Material

Supplementary Figure 1 Spearman correlation analysis between the risk score and clinical features, including sex **(A)**, age **(B)**, grade **(C)**, pathological stage **(D)**, T stage **(E)**, N stage **(F)**, and M stage **(G)**. Sex, age, stage, and risk score were all found to be independent predictive variables in the univariate regression analysis **(H)**. Age, stage, and risk score were all found to be independent predictive variables in the multivariate regression analysis **(D)**.

Supplementary Figure 2 Enriched gene sets in the KEGG pathway in the high- **(A)** and low-risk patients **(B)**. Enriched gene sets in the GO pathway in the high- **(C)** and low-risk patients **(D)**.
