# Supplementary figures and images for: A Signature Based on Costimulatory Molecules for the Assessment of Prognosis and Immune Characteristics in Patients With Stomach Adenocarcinoma
Source: Front Immunol. 2022 Jul 22;13:928742. doi: 10.3389/fimmu.2022.928742 (PMC9353527; doi:10.3389/fimmu.2022.928742)

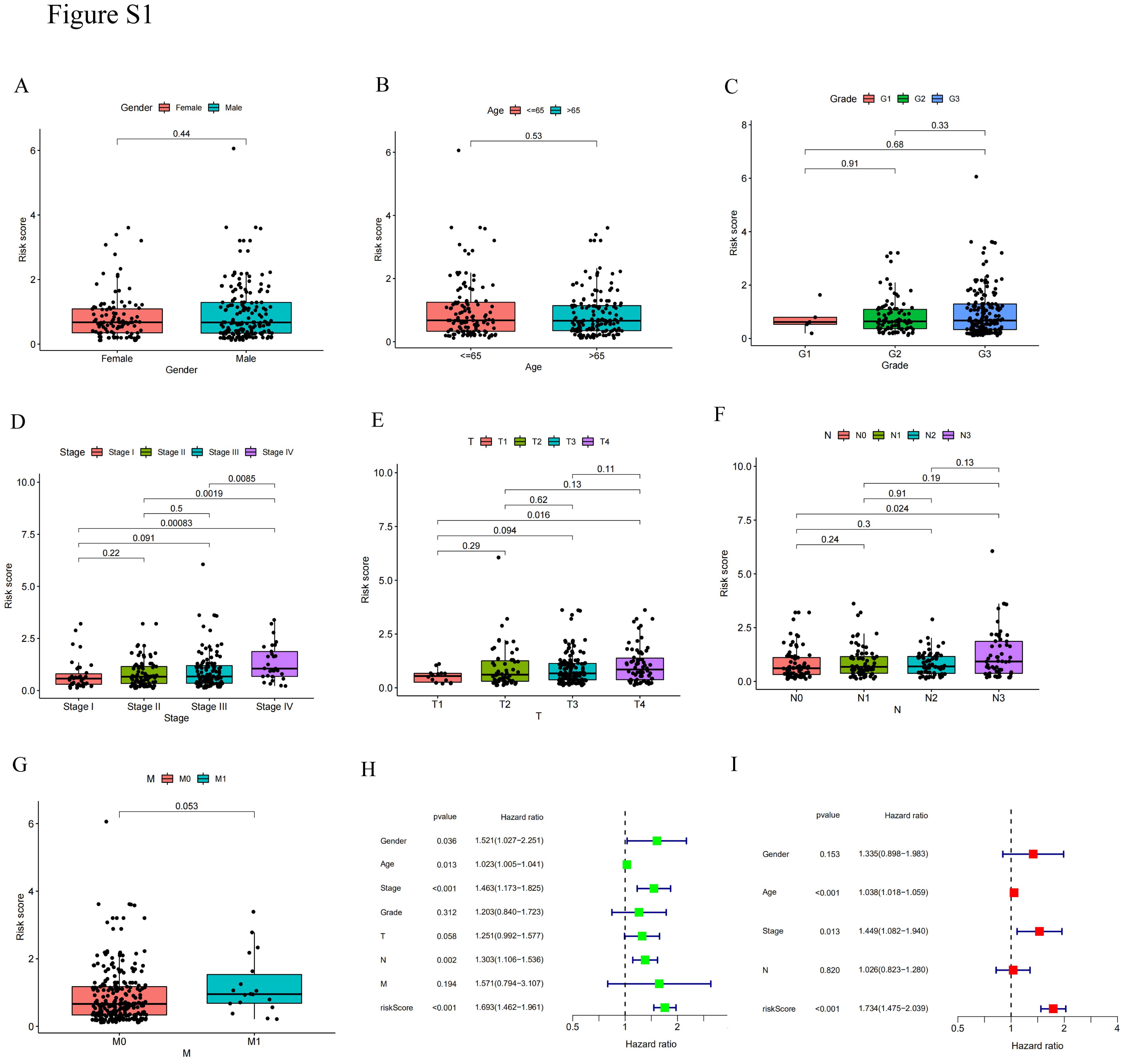

Supplement: Supplementary Figure 1 — Spearman correlation analysis between the risk score and clinical features, including sex (A), age (B), grade (C), pathological stage (D), T stage (E), N stage (F), and M stage (G). Sex, age, stage, and risk score were all found to be independent predictive variables in the univariate regression analysis (H). Age, stage, and risk score were all found to be independent predictive variables in the multivariate regression analysis (D). [file Image_1.jpeg]

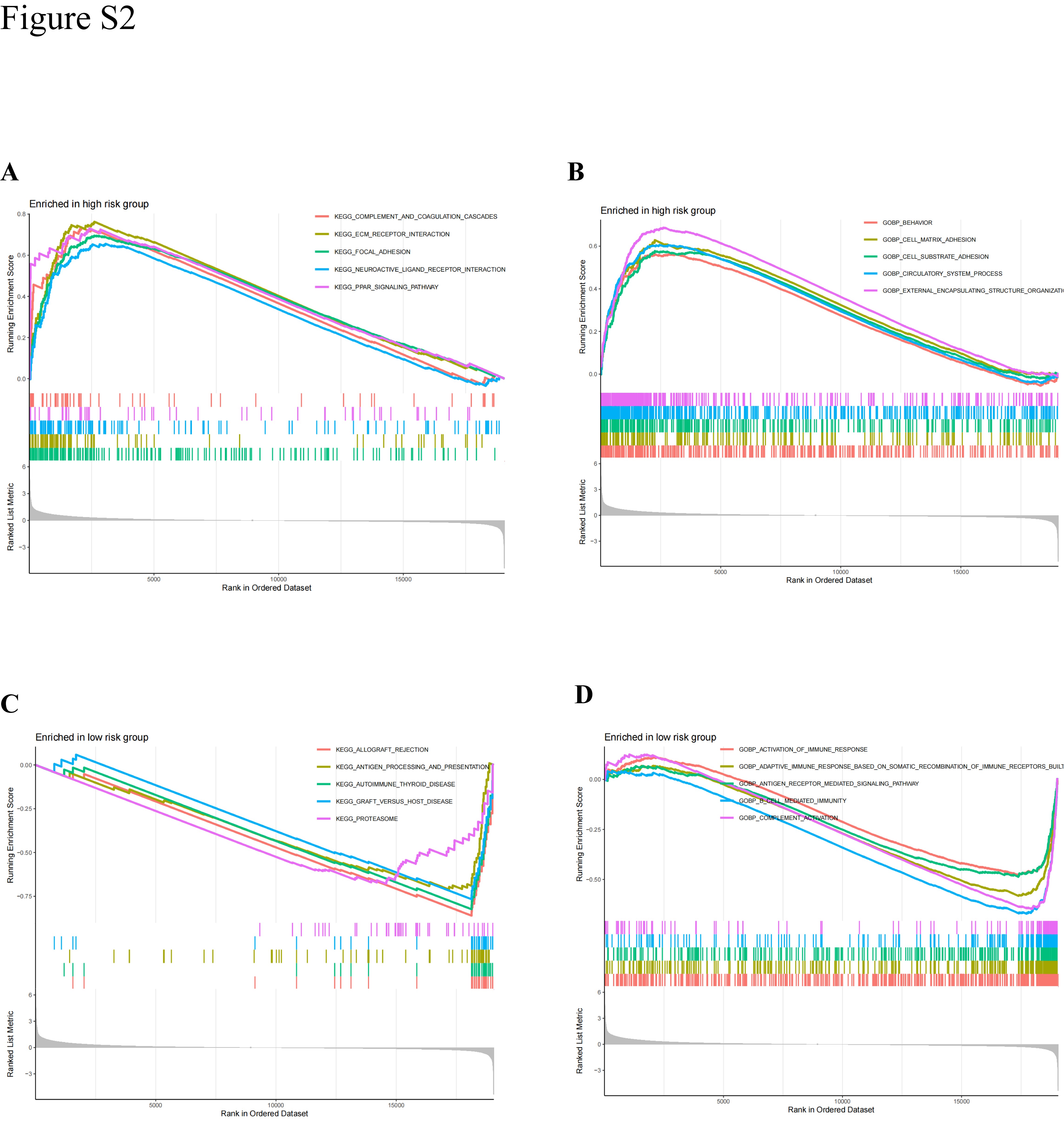

Supplement: Supplementary Figure 2 — Enriched gene sets in the KEGG pathway in the high- (A) and low-risk patients (B). Enriched gene sets in the GO pathway in the high- (C) and low-risk patients (D). [file Image_2.jpeg]

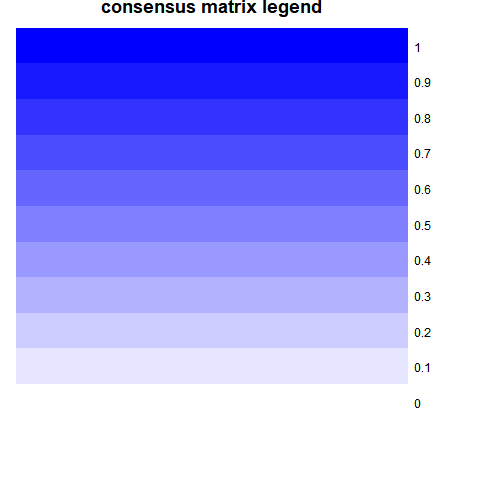

Supplement: Supplementary file 4 [file DataSheet_2.zip › Raw date/2.Consensus score/consensus001.png]

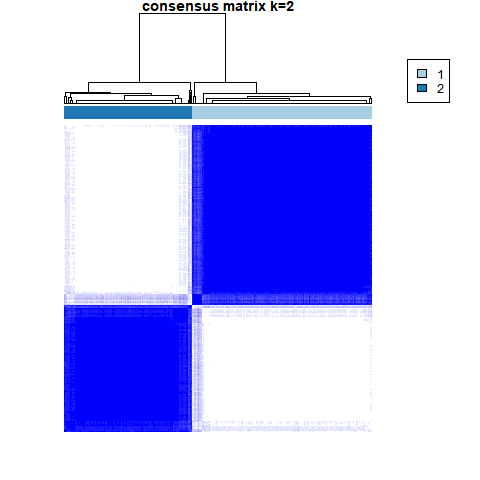

Supplement: Supplementary file 4 [file DataSheet_2.zip › Raw date/2.Consensus score/consensus002.png]

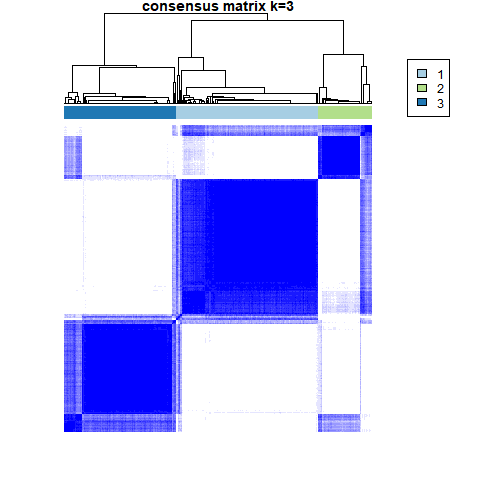

Supplement: Supplementary file 4 [file DataSheet_2.zip › Raw date/2.Consensus score/consensus003.png]

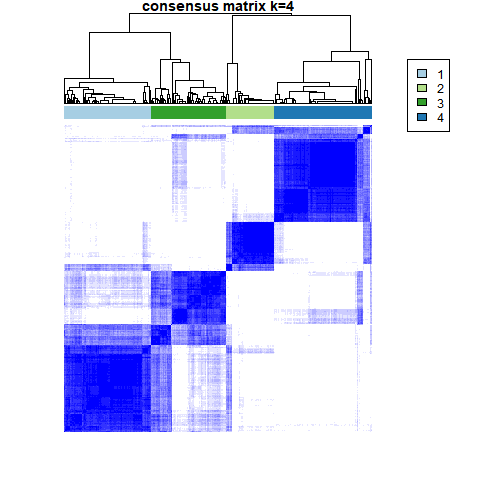

Supplement: Supplementary file 4 [file DataSheet_2.zip › Raw date/2.Consensus score/consensus004.png]

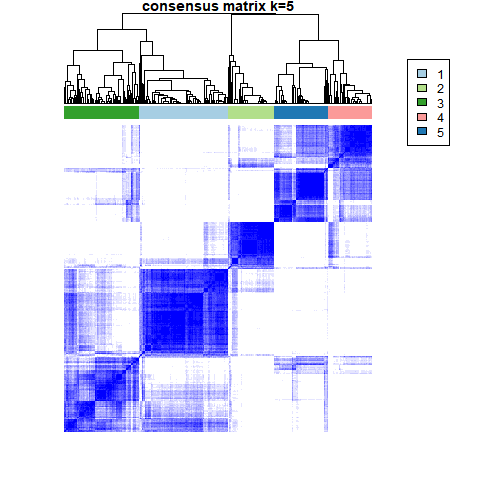

Supplement: Supplementary file 4 [file DataSheet_2.zip › Raw date/2.Consensus score/consensus005.png]

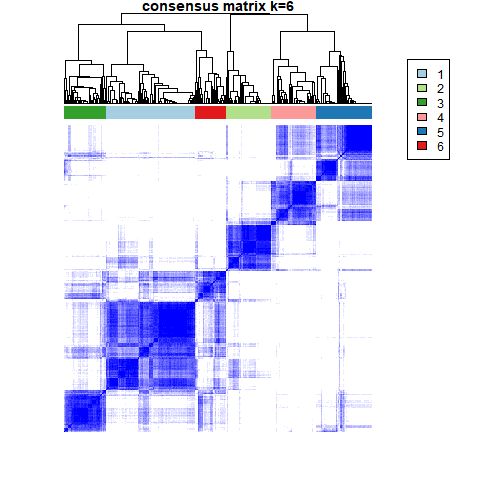

Supplement: Supplementary file 4 [file DataSheet_2.zip › Raw date/2.Consensus score/consensus006.png]

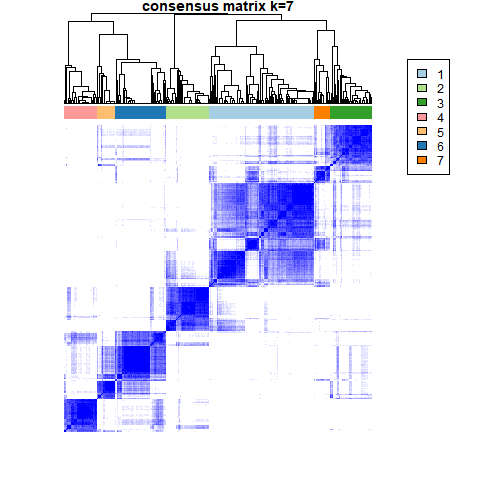

Supplement: Supplementary file 4 [file DataSheet_2.zip › Raw date/2.Consensus score/consensus007.png]

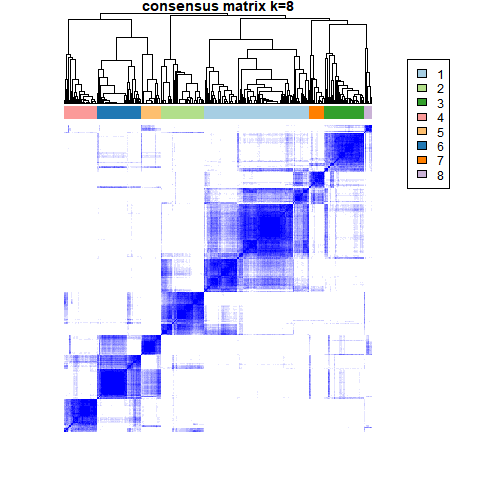

Supplement: Supplementary file 4 [file DataSheet_2.zip › Raw date/2.Consensus score/consensus008.png]

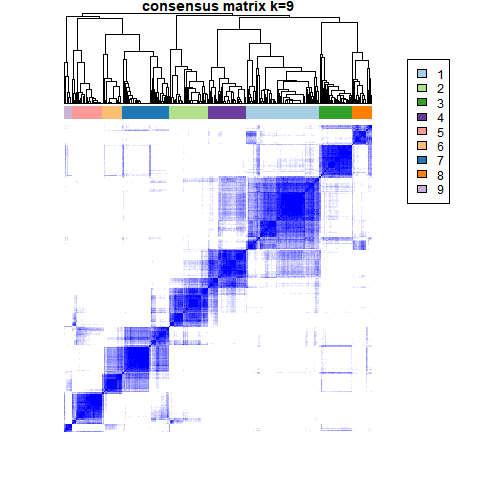

Supplement: Supplementary file 4 [file DataSheet_2.zip › Raw date/2.Consensus score/consensus009.png]

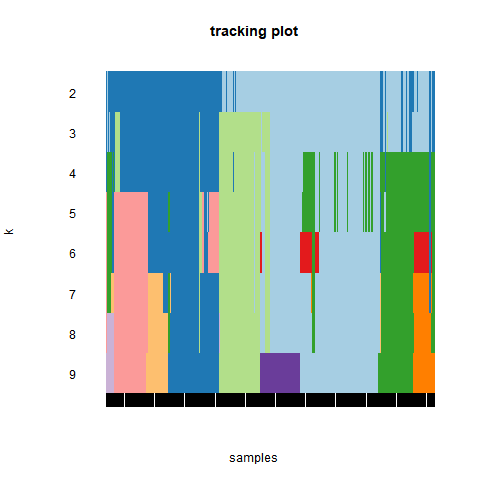

Supplement: Supplementary file 4 [file DataSheet_2.zip › Raw date/2.Consensus score/consensus012.png]

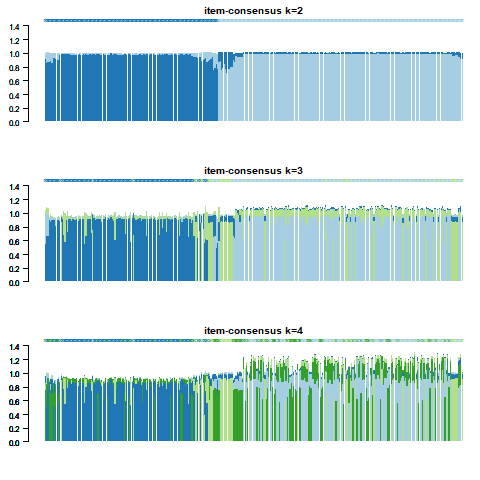

Supplement: Supplementary file 4 [file DataSheet_2.zip › Raw date/2.Consensus score/icl001.png]

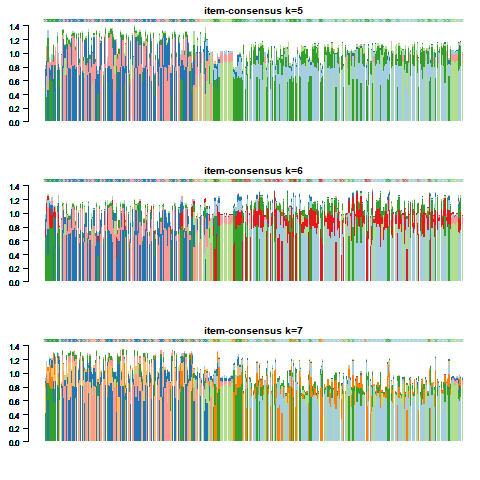

Supplement: Supplementary file 4 [file DataSheet_2.zip › Raw date/2.Consensus score/icl002.png]

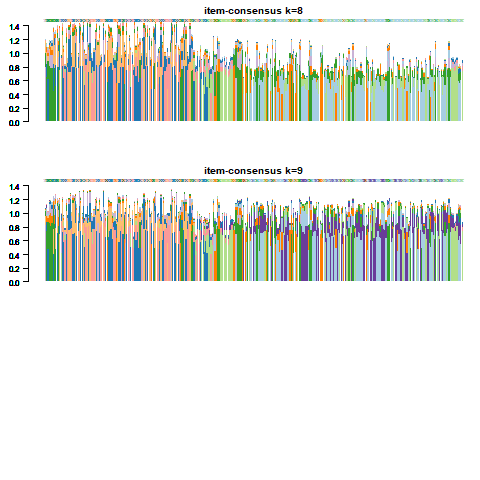

Supplement: Supplementary file 4 [file DataSheet_2.zip › Raw date/2.Consensus score/icl003.png]

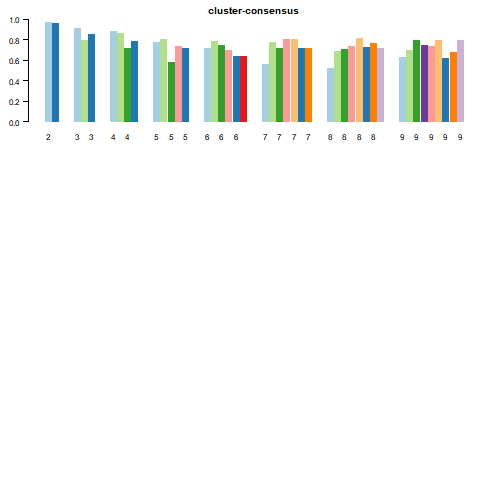

Supplement: Supplementary file 4 [file DataSheet_2.zip › Raw date/2.Consensus score/icl004.png]
